# Thymocid^®^, a Standardized Black Cumin (*Nigella sativa*) Seed Extract, Modulates Collagen Cross-Linking, Collagenase and Elastase Activities, and Melanogenesis in Murine B16F10 Melanoma Cells

**DOI:** 10.3390/nu12072146

**Published:** 2020-07-19

**Authors:** Huifang Li, Nicholas A. DaSilva, Weixi Liu, Jialin Xu, George W. Dombi, Joel A. Dain, Dongli Li, Jean Christopher Chamcheu, Navindra P. Seeram, Hang Ma

**Affiliations:** 1 School of Biotechnology and Health Sciences, Wuyi University, International Healthcare Innovation Institute (Jiangmen), Jiangmen 529020, China; huifang_li@uri.edu (H.L.); wyuchemldl@126.com (D.L.); 2 Bioactive Botanical Research Laboratory, Department of Biomedical and Pharmaceutical Sciences, College of Pharmacy, University of Rhode Island, Kingston, RI 02881, USA; NickDasilva91@gmail.com (N.A.D.); jialin_xu@mail.neu.edu.cn (J.X.); nseeram@uri.edu (N.P.S.); 3 Department of Chemistry, University of Rhode Island, Kingston, RI 02881, USA; weixi_liu@my.uri.edu (W.L.); gdombi@chm.uri.edu (G.W.D.); jdain@chm.uri.edu (J.A.D.); 4 Institute of Biochemistry and Molecular Biology, College of Life and Health Sciences, Northeastern University, Shenyang 110819, China; 5 School of Basic Pharmaceutical and Toxicological Sciences, College of Pharmacy, University of Louisiana at Monroe, Monroe, LA 71209, USA; chamcheu@ulm.edu

**Keywords:** black cumin, *Nigella sativa*, Thymocid^®^, skin aging, glycation, collagen, collagenase, elastase, melanogenesis, cosmeceutical

## Abstract

Black cumin (*Nigella sativa*) seed extract has been shown to improve dermatological conditions, yet its beneficial effects for skin are not fully elucidated. Herein, Thymocid^®^, a chemically standardized black cumin seed extract, was investigated for its cosmeceutical potential including anti-aging properties associated with modulation of glycation, collagen cross-linking, and collagenase and elastase activities, as well as antimelanogenic effect in murine melanoma B16F10 cells. Thymocid^®^ (50, 100, and 300 µg/mL) inhibited the formation of advanced glycation end-products (by 16.7–70.7%), collagen cross-linking (by 45.1–93.3%), collagenase activity (by 10.4–92.4%), and elastases activities (type I and III by 25.3–75.4% and 36.0–91.1%, respectively). In addition, Thymocid^®^ (2.5–20 µg/mL) decreased melanin content in B16F10 cells by 42.5–61.6% and reduced cellular tyrosinase activity by 20.9% (at 20 µg/mL). Furthermore, Thymocid^®^ (20 µg/mL for 72 h) markedly suppressed the mRNA expression levels of melanogenesis-related genes including microphthalmia-associated transcription factor (*MITF*), tyrosinase-related protein 1 (*TYRP1*), and *TYRP2* to 78.9%, 0.3%, and 0.2%, respectively. Thymocid^®^ (10 µg/mL) also suppressed the protein expression levels of MITF (by 15.2%) and TYRP1 (by 97.7%). Findings from this study support the anti-aging and antimelanogenic potential of Thymocid^®^ as a bioactive cosmeceutical ingredient for skin care products.

## 1. Introduction

*Nigella sativa* Linn. (family Ranunculaceae), commonly known as black seed, black cumin, or cumin noir, originated in regions of Eastern Europe, the Middle East, northern Africa, the Indian subcontinent, and the west and middle of Asia [1]. In these regions, it has a long history of use for culinary purpose as a spice, natural seasoning, or flavoring, as well as for medicinal purposes in traditional folk medicine systems to treat a variety of ailments [2]. Black cumin seeds are recognized for their great nutritional value as a source of nutrients including iron, copper, zinc, phosphorus, calcium, thiamin, niacin, pyridoxine, and folic acid [3]. In addition, black cumin seeds are a good source of plant-based proteins as they have been reported to have a high index of net protein utilization, protein efficiency ratio, and net dietary protein energy percent [3]. Moreover, phytochemical investigations of black cumin seeds revealed that thymoquinone (TQ) and its derivatives including thymohydroquinone, dithymoquinone, thymol, and carvacrol are the major chemicals in black cumin seed oil [4]. In addition, other phytochemicals, such as alkaloids including nigellicine, nigellimine, nigellidine, 17-*O*-(β-d-glucopyranosyl)-4-*O*-methylnigellidine, 4-*O*-methylnigellidine, nigelanoid, nigeglanine, and 4-*O*-methylnigeglanine have been identified as minor constituents of black cumin seed oil [4,5,6]. Apart from their nutritional values, black cumin seed extracts (BCSEs) have been reported to display diverse biological and pharmacological activities including antioxidant, anti-inflammatory, anticarcinogenic and antimutagenic, antidiabetic, antimicrobial, and immunological effects [7,8,9,10]. Moreover, pre-clinical and clinical studies have reported efficacious activities of BCSEs on dermatological conditions. Several in vitro studies demonstrated that protective effects of a BCSE on cutaneous disorders could be attributed to its antimicrobial effects including antibacterial, antiviral, antifungal, and antiparasitic activity [11]. BCSE’s antimicrobial effects have also been studied in a clinical trial, where a treatment with 10% oil lotion of BCSE for 2 months exerted an anti-acne effect via reduction in the mean lesion count of papules and pustules [12]. However, only limited number of studies have reported BCSEs’ cosmeceutical applications, such as its modulatory effects on the production of melanin in the melanophores from wall lizard (*Podarcis muralis*), which was attributed to the stimulation of cholinergic receptors [13]. However, to date, the cosmeceutical effects of BCSE as an antiwrinkle/-aging agent, have not yet been reported.

Skin wrinkling is a natural and observable index of the process of aging. It can be exacerbated by a combination of external oxidative stress (e.g., ultraviolet light radiation and pollutants) and endogenous factors (e.g., protein glycation and degradation). Skin wrinkles are formed when the physiological structure of skin tissue is impaired. Skin structure is collectively maintained by a group of connective and supportive proteins including collagen, elastin, claudin, laminin, nidogen, occludin, zonula occludens, and junctional adhesion molecule [14,15,16,17,18]. Amongst these proteins, collagen and elastin are major supportive molecules for the structure of skin tissue, and their structural damage and degradation are directly associated with the formation of skin wrinkles [16]. Collagen is a long-lasting protein with a half-life of over a decade and is subject to chronic internal stress such as glycation. The formation of advanced glycation end-products (AGEs) leads to the alteration of protein structure and the generation of free radicals, which further results in the impairment of the protein’s physiological functions including its ability of maintaining the structure of skin tissue. Protein degradation is another crucial contributing factor to the formation of skin wrinkles [14]. Elastin, an elastic protein that maintains skin structure following stretching or contraction, can be degraded by a protease enzyme known as elastase [19]. Therefore, antiglycation agents and inhibitors of collagenase and elastase from dietary natural products have attracted immense research interest for the management of skin aging [20]. However, the protective effects of BCSEs against collagen and elastin degradation remain unclear.

Over the past decade, our laboratory has conducted phytochemical and biological investigations of dietary natural products including extracts of medicinal plants and functional foods. During the course of our studies, several botanical extracts exhibited promising anti-aging and beneficial effects for skin [21,22,23]. Among these herbal extracts, a BCSE was investigated for its phytochemical constituents and biological effects. This led to the identification of TQ (as the major phytochemical of this BCSE) and other bioactive compounds such as indazole-type alkaloids with antihyperglycemic effects [6]. A proprietary BCSE, namely, Thymocid^®^, is commercially produced by a cold compressed method without the use of extraction solvents. It contains omega-6 fatty acids such as linoleic acid and is chemically standardized to TQ content. As part of our group’s continuing research efforts to study the beneficial effects to skin of bioactive dietary ingredients, the current study was designed to evaluate the cosmeceutical properties of Thymocid^®^ with in vitro enzymatic and cell-based assays for (1) effects on glycation of bovine serum albumin and collagen cross-linking; (2) anticollagenase and anti-elastase activities; (3) effects on melanogenesis in murine melanoma B16F10 cells; and, (4) effects on the expressions of melanogenesis-related genes and proteins in B16F10 cells.

## 2. Materials and Methods

### 2.1. Chemicals

Thymoquinone (TQ), 3-(4,5-dimethylthiazol-2-yl)-5-(3-carboxymethoxyphenyl)-2-(4-sulfenyl)-2-H-tetrazolium salt (MTS), methanol (analytical grade), trifluoroacetic acid, Tris-HCl buffer (pH 10), phosphate buffer saline (PBS, pH 7.4), kojic acid, aminoguanidine (AG), epigallocatechin gallate (EGCG), L-tyrosine, L-3,4-dihydroxyphenylalanine (L-DOPA), N-succinyl-Ala-Ala-ala-p-nitroanilide (AAAPVN), mushroom tyrosinase, elastase enzymes (type Ⅰ and III) from porcine pancreas, bovine serum albumin (BSA), methylglyoxal (MGO), 3-(4,5-dimethylthiazol-2-yl)-5-(3-carboxymethoxyphenyl)-2-(4-sulfophenyl)-2H-tetrazolium (MTS) reagent, Triton X-100 agent, and sodium azide were purchased from Sigma-Aldrich Co. (St. Louis, MO, USA). Bovine type I collagen was obtained from Advanced BioMatrix Inc. (PureCol^®^, Catalog #5005; Carlsbad, CA, USA). A commercially available black cumin seed extract (Thymocid^®^) was kindly provided by Verdure Sciences (Noblesville, IN, USA).

### 2.2. Quantification of Thymoquinone (TQ) in Thymocid^®^

The level of TQ in Thymocid^®^ was quantified by using the high-performance liquid chromatography (HPLC) method with an Hitachi HPLC instrument (Hitachi Instruments, Inc., San Jose, CA, USA), an Alltima C_18_ column (250 × 4.6 mm i.d., 5 µm), and a solvent system consisting of 0.1% trifluoroacetic acid in water (A) and methanol (B). A linear gradient eluting method was used as follows: 0–20 min, 50–100% B; 20–21 min, 100–50% B; 20–28 min, 50 % B with a total run time of 28 min, a flow rate of 0.75 mL/min, and an injection volume of 10 µL. Thymocid^®^ was dissolved in dimethyl sulfoxide (DMSO) at various concentrations (0.1–4 mg/mL) and monitored at a range of wavelengths from 200 to 400 nm with a photodiode array detector (see Supplementary Materials Figure S1). A standard curve of TQ at various concentrations (1–200 µg/mL) monitored at the wavelength of 254 nm, which is the characteristic wavelength for TQ, was constructed for its quantification in Thymocid^®^ (see Supplementary Materials Figure S2).

### 2.3. Bovine Serum Albumin (BSA)–Fructose Glycation Assay

The antiglycation assay was performed according to previously reported methods using a BSA–fructose model [24]. Briefly, a BSA–fructose reaction mixture containing BSA (10 mg/mL) and D-fructose (100 mM) was prepared in phosphate buffer saline (PBS; 0.2 M, pH 7.2). Next, different concentrations of Thymocid^®^ (50, 100, and 300 µg/mL) were added to the BSA–-fructose mixture and incubated at 37 °C on a shaking rack for a duration of 14 days. Aminoguanidine (AG) was used as a positive control. The formation of AGEs was monitored by the measurement of intrinsic fluorescence at excitation and emission wavelengths of 340 and 435 nm, respectively, using a plate reader.

### 2.4. Collagen Cross-Linking Assay

A reaction mixture consisting of bovine type I collagen (1.5 mg/mL), Thymocid^®^ (at 50, 100, and 300 µg/mL), methylglyoxal (MGO; 5 mM), and sodium azide (10 mM) in PBS was incubated at 37 °C for 30 days. The reaction mixture of each sample (200 µL) was then transferred to a 96-well black fluorescence reading plate. The level of cross-linked collagen was monitored by measuring the fluorescent intensity of each well with excitation and emission wavelengths at 340 and 435 nm, respectively, using a plate reader.

### 2.5. Collagenase Inhibition Assay

A collagenase inhibition assay was conducted using a collagenase activity assay kit (Abcam Inc., Cambridge, MA, USA) following the manufacturer’s instructions. Briefly, assay buffer (180 µL) and a mixture of test sample at various concentrations or a positive control, 1,10-phenanthroline (10 µL), and collagenase solution (10 µL) were incubated in a 96-well plate at 37 °C for 15 min. The substrate N-(3-[2-furyl]-acryloyl)-Leu-Gly-Pro-Ala (FALGPA) solution (100 µL) was then added to each well. The absorbance of each well was recorded at a wavelength of 345 nm with a kinetic mode for 30 min using a plate reader. The activity of collagenase was calculated as follow: collagenase activity = (∆ODc/∆T) × 0.2/(0.53 × V), where ∆ODc = difference of optical density (OD) reading from sample at different time points, 0.2 = reaction volume (mL), 0.53 = millimolar extinction coefficient of FALGPA, V = sample volume added into the reaction well (mL). The inhibition rate was calculated as inhibition% = 100 × (Activity_Enzyme_ − Activity_Sample_)/Activity_Enzyme_%.

### 2.6. Elastase Inhibition Assay

An elastase inhibition assay was conducted using previously reported methods with minor modifications [25]. Briefly, a mixture of test samples (10 µL), elastase solution (20 µL; 0.5 U/mL), and Tris-HCl buffer (140 µL; 2 mM; pH 8.0) were incubated in a 96-well plate at room temperature for 15 min. Then, substrate AAAPVN solution (50 µL; 1 mg/mL) in Tris-HCL buffer was added to each well, and the reaction mixtures were allowed to incubate at room temperature for 1 min. The absorbance of each well was recorded at a wavelength of 410 nm using a plate reader.

### 2.7. Tyrosinase Inhibition Assay

A tyrosinase inhibition assay was conducted as per previously reported methods with slight modifications [26]. In brief, a mixture of test samples (40 µL) and mushroom tyrosinase solution (100 U/mL) in PBS (0.1 M; 120 µL) was incubated in wells of a 96-well plate at room temperature for 15 min. Following incubation, L-tyrosine solution (2.5 mM; 40 µL) or L-DOPA solution (2.5 mM; 40 µL) in PBS was added to each well, and the reaction mixtures were incubated at 37 ℃ for 30 min. The absorbance of each well was recorded at a wavelength of 490 nm using a plate reader.

### 2.8. Cell Culture

Murine melanoma B16F10 cells obtained from American Type Culture Collection (ATCC, Rockville, MD, USA) were cultured as recommended by ATCC. Briefly, B16F10 cells were grown in Dulbecco’s modified Eagle’s medium (DMEM; Life Technologies, Gaithersburg, MD, USA) supplemented with 10% fetal bovine serum (Life Technologies) and 1% antibiotic solution (Sigma-Aldrich Co., St. Louis, MO, USA). Cells were maintained at 37 °C in the presence of 5% CO_2_ and constant humidified atmosphere. Test samples were dissolved in DMSO as stock solution and then diluted with cell culture medium to the desired concentrations (DMSO < 0.1%).

### 2.9. Cell Viability Assay

The viability of B16F10 cells was determined by the MTS assay as described previously with minor modifications [27]. Briefly, cells were seeded in 96-well plates at a density of 5 × 10^3^ cells per well and allowed to attach overnight. Next, the culture medium was replaced with fresh medium supplemented with various concentrations of Thymocid^®^ (2.5, 5, 10, 20, and 40 µg/mL) for 72 h. After the incubation, freshly prepared MTS reagent (20 µL) was added to each well and incubated at 37 °C for 30 min, and optical density of each well was measured at a wavelength of 490 nm using a plate reader.

### 2.10. Melanogenesis Assay

The antimelanogenic effect of Thymocid^®^ was evaluated by the measurement of melanin content in B16F10 cells following previously reported method with modifications [27]. Briefly, cells were seeded in 96-well plates at a density of 5 × 10^3^ cells per well and allowed to attach for 24 h. Cells were then treated with Thymocid^®^ at concentrations of 2.5, 5, 10, and 20 µg/mL for 72 h. Then cells were lysed by adding sodium hydroxide solution (0.1 M; 1 mL), and lysed cells were centrifuged (3000× *g* for 5 min). The supernatant was decanted, and the cell pellet was exposed to sodium hydroxide (200 µL), followed by placing in a water bath at 80 °C for 1 h. Samples were then briefly mixed by vortex, and melanin content was transferred to a 96-well plate. The absorbance of each well was measured at a wavelength of 405 nm using a plate reader.

### 2.11. Cellular Tyrosinase Activity Assay

The cell-based tyrosinase assay was performed using a previously reported method with modifications [28]. Briefly, B16F10 cells were seeded at 4 × 10^4^ cells per well in a 24-well plate and allowed to grow for 24 h prior to being treated with Thymocid^®^ (2.5, 5, 10, and 20 µg/mL) for 72 h. Next, cells were harvested and washed twice with ice-cold PBS followed by centrifugation at 12,000× *g* for 10 min, and cells pellets were re-suspended in PBS containing Triton X-100 (1%). The cells were lysed by a freeze-and-thaw cycle to release tyrosinase from the melanosome membrane. Cellular tyrosinase was collected by centrifugation at 10,000× *g* at 4 °C for 30 min. A reaction mixture consisting of cellular tyrosinase (20 μg in 200 µL of PBS) and L-DOPA solution (1.25 mM) was incubated at 37 °C for 30 min. The formation of dopachrome was quantified by measuring the optical density at a wavelength of 495 nm using a plate reader.

### 2.12. Real-Time Polymerase Chain Reaction (RT-PCR)

The mRNA expression level of melanin-synthesis-related genes including microphthalmia-associated transcription factor (*MITF*), tyrosinase (*TYR*), tyrosinase-related protein-1 (*TYRP1*), and tyrosinase-related protein-2 (*TYRP2*) were measured by a real-time polymerase chain reaction (RT-PCR) assay with a previously reported method [27]. Briefly, B16F10 cells were seeded in 6-well plates at a density of 1 × 10^5^ cells per well and allowed to grow for 24 h. Then cells were treated with Thymocid^®^ (2.5 or 10 µg/mL) for 24, 48, or 72 h. Total RNA was isolated from cells using TRIzol reagent (Invitrogen, Carlsbad, CA, USA) according to the manufacturer’s instructions. The extracted genes were quantified by using a SYBR Green kit (Thermo Fisher Scientific, Grand Island, NY, USA) and compared to levels of *b2m* rRNA as a reference housekeeping gene.

### 2.13. Preparation of Cellular Protein Lysates and Western Blot Assay

B16F10 cells were seeded in 6-well plates at a density of 1.0 × 10^5^ cells per well and allowed to grow for 24 h and were then treated with Thymocid^®^ (2.5 and 10 µg/mL) and cultured for 72 h. After washing with PBS, B16F10 cells were harvested, whole-cell lysates were prepared and quantified, and the protein expressions of MITF, TYR, TYRP1, and TYRP2 were quantified by Western blot assay, as described previously [27]. Antibodies including anti-MITF (ab3201), anti-TYRP1 (ab178676), anti-TYRP2 (ab103463), and anti-β-Actin antibody (ab8227) were obtained from Abcam, Cambridge, MA, USA. The western blot (WB) bands were detected on X-ray films using an enhanced chemiluminescence (ECL) detection kit (GE Healthcare, Piscataway, NJ, USA) according to the manufacturer’s protocol.

### 2.14. Statistical Analyses

Data are presented as mean ± standard deviation (S.D.) of at least three replicated experiments. Two-tailed unpaired Student’s *t* test or ANOVA with Tukey post-test was used for statistical analysis of the data using the GraphPad Prism software 6.0 or Office Excel 2010 software. Significance for all tests was defined as *p* ≤ 0.05 (*), *p* ≤ 0.01 (**), *p* ≤ 0.001 (***), and *p* ≤ 0.0001 (****).

## 3. Results

### 3.1. Thymocid^®^ Inhibits the Formation of Advanced Glycation End-Products (AGEs) and Collagen Cross-Linking

**Figure 1:**
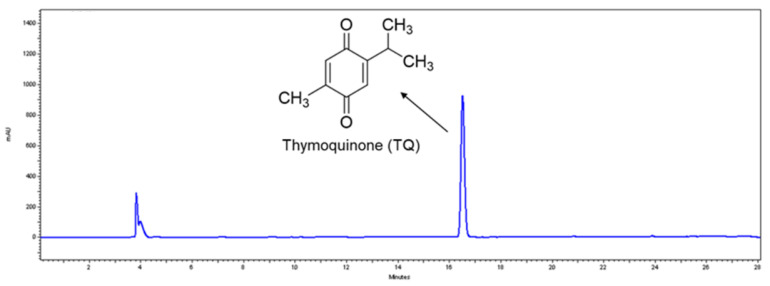
Chemical structure of thymoquinone (TQ) and HPLC profile of a black cumin seed extract (Thymocid^®^) standardized to TQ content.

First, a commercially available BCSE, Thymocid^®^, was standardized to thymoquinone (TQ), as its major phytochemical marker. The level of TQ in Thymocid^®^ was quantified using a standard curve based on the HPLC analysis (see Supplementary Materials Figure S2) and was determined to be 5.12% (Figure 1).

**Figure 2:**
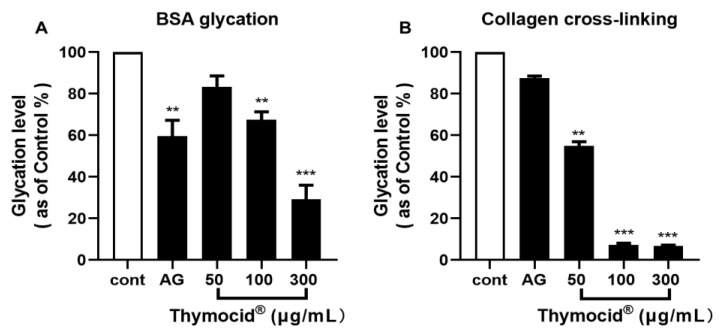
Effects of Thymocid^®^ on the formation of advanced glycation end-products (AGEs) in two glycation models including bovine serum albumin (BSA)–fructose (**A**) and type I collagen cross-linking (**B**). The inhibitory effects of Thymocid^®^ (50, 100, and 300 µg/mL) and aminoguanidine (AG) (a positive control) on the formation of AGEs and level of protein cross-linking were determined by fluorescent assays. Values are expressed in means ± standard deviation (S.D.) from three experiment replicates. Significance was defined as ** *p* < 0.01 and *** *p* < 0.001 when compared to the control group.

Next, Thymocid^®^ was assessed for the anti-skin-aging effects by measuring its inhibitory effects on the formation of advanced glycation end-products (AGEs) using a BSA model and protein cross-linking using a type I collagen model. Thymocid^®^ (at 50, 100, and 300 µg/mL) reduced the fructose-induced formation of AGEs by 16.7%, 32.5%, and 70.7%, respectively (Figure 2A), in a concentration-dependent manner. Aminoguanidine (AG; employed as a positive control) had an inhibition rate of 59.6% at 100 µg/mL. In addition, Thymocid^®^ (at 50, 100, and 300 µg/mL) inhibited methylglyoxal (MGO)-induced collagen cross-linking by 45.1%, 92.6%, and 93.3%, respectively (Figure 2B), whereas, AG (at 100 µg/mL) was less active with an inhibition rate of 12.5%.

### 3.2. Thymocid^®^ Inhibits Collagenase Activity

**Figure 3:**
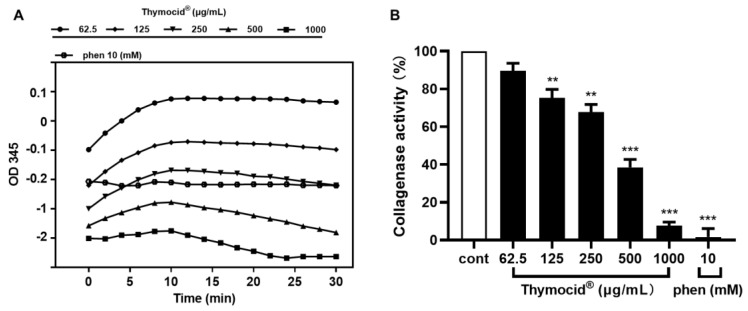
The optical density at a wavelength of 345 nm (OD_345_) values of collagenase enzyme treated with Thymocid^®^ or a positive control phen (1,10-phenanthroine), over 30 min in a kinetic mode (**A**). The inhibitory effect of Thymocid^®^ (62.5–1000 µg/mL) and phen (10 mM) on collagenase activity was measured by a colorimetric assay (**B**). The inhibition rates are expressed as mean ± S.D. from three replicated experiments. Significance was defined as ** *p* < 0.001 and *** *p* < 0.0001 when compared to the control group.

The effect of Thymocid^®^ on collagen degradation was evaluated by assessing the inhibitory effects on collagenase enzyme activity. The OD_345_ values of enzymes treated with 1,10-phenanthroine (phen), a known inhibitor ( employed as a positive control), was observed to decrease over 30 min, while Thymocid^®^ (at 62.5–1000 µg/mL) decreased the OD_345_ values within 10–30 min, suggesting that both 1,10-phenanthroine and Thymocid^®^ reduced the activity of collagenase (Figure 3A). Thymocid^®^ (at 62.5–1000 µg/mL) reduced collagenase activity by 10.4–92.4%, respectively (Figure 3B), while phen (at 10 mM) showed an inhibition rate of 98.4%.

### 3.3. Thymocid^®^ Inhibits Elastase Activity

Thymocid^®^ was further evaluated for its antiwrinkle property by measuring its inhibitory effects on the activity of elastases (type I and III). Thymocid^®^ (at concentrations of 62.5–1000 µg/mL) inhibited elastase activities in a concentration-dependent manner as it reduced the activity of type I and III elastase by 25.3–75.4% and 36.0–91.1%, respectively (Table 1). Epigallocatechin gallate (EGCG; 92 µg/mL), used as a positive control, provided an inhibition rate of 73.0% and 75.2% on type I and III elastase, respectively. It should be noted that TQ, the major phytochemical in Thymocid^®^, also reduced the activities of collagenase and elastases (type I and III) by 18.0–30.0%, 16.1–30.2%, and 12.9–45.3%, at concentrations of 62.5, 125, 250, 500, and 1000 µg/mL, respectively (see Supplementary Materials Tables S1 and S2).

### 3.4. Thymocid^®^ Increases Tyrosinase Activity

We further evaluated the effects of Thymocid^®^ in melanin-biosynthesis-related bioassays. First, Thymocid^®^ was evaluated for its modulatory effect on tyrosinase activity using two enzyme substrates including L-tyrosine and L-DOPA. As shown in Table 2, when tested with the different substrates, Thymocid^®^ (62.5–1000 µg/mL) dose-dependently increased the activity of tyrosinase to 153.3–228.7% for L-tyrosine as substrate, and 113.7–146.3% for L-DOPA as substrate, respectively (Table 2). Kojic acid (at 10 µg/mL), a known tyrosinase inhibitor, was included as a positive control and it reduced tyrosinase activity to 49.5% and 66.1%, respectively. In addition, TQ (at 62.5, 125, 250, 500, and 1000 µg/mL) also increased the activity of tyrosinase to 120.7–110.8% and 100.8–117.7%, respectively, when assayed with substrates L-tyrosine and L-DOPA (see Supplementary Materials Table S3).

### 3.5. Thymocid^®^ Reduces the Melanin Content in B16F10 Melanoma Cells

**Figure 4:**
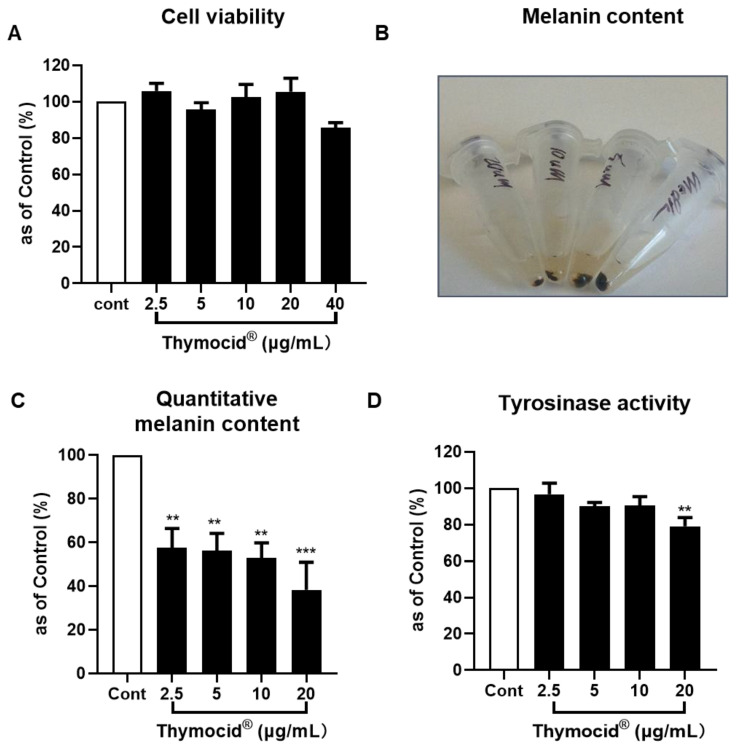
Effects of Thymocid^®^ (2.5, 5, 10, and 20 µg/mL) on the cell viability of murine melanoma B16F10 cells (**A**), melanin content (**B**), and its quantification (**C**) in B16F10 cells cultured with Thymocid^®^ for 72 h, and cellular tyrosinase activity in B16F10 cells treated with Thymocid^®^ for 72 h (**D**). Significance was defined as ** *p* < 0.01 and *** *p* < 0.001 when compared to the control group and values are presented as the means ± S.D. from three experiment replicates.

To further evaluate whether Thymocid^®^ can modulate the production of melanin, cell-based assays were conducted in murine melanoma B16F10 cells. The cytotoxicity of Thymocid^®^ (2.5, 5, 10, 20, and 40 µg/mL) on B16F10 cells was evaluated by measuring cell viability using the MTS assay. Thymocid^®^ was nontoxic to B16F10 cells at concentrations ranging from 2.5 to 20 µg/mL, as it maintained the viability of B16F10 cells greater than 95.7% (Figure 4A), and these nontoxic concentrations were selected for further bioassays. Next, the antimelanogenic effect of Thymocid^®^ in B16F10 cells was evaluated. We observed that Thymocid^®^ (2.5, 5, 10, and 20 µg/mL) suppressed the production of melanin (Figure 4B) to 57.5%, 56.1%, 52.9%, and 38.4%, respectively, as compared to the control group without treatment of Thymocid^®^ (Figure 4C). Furthermore, we assessed the effect of Thymocid^®^ on cellular tyrosinase activity in B16F10 cells. Compared to the control group, Thymocid^®^ reduced cellular tyrosinase activity by 20.9% at the highest tested concentration (20 µg/mL) (Figure 4D).

### 3.6. Thymocid^®^ Suppresses the mRNA and Protein Expression Levels of Melanogenesis-Related Markers in B16F10 Cells

**Figure 5:**
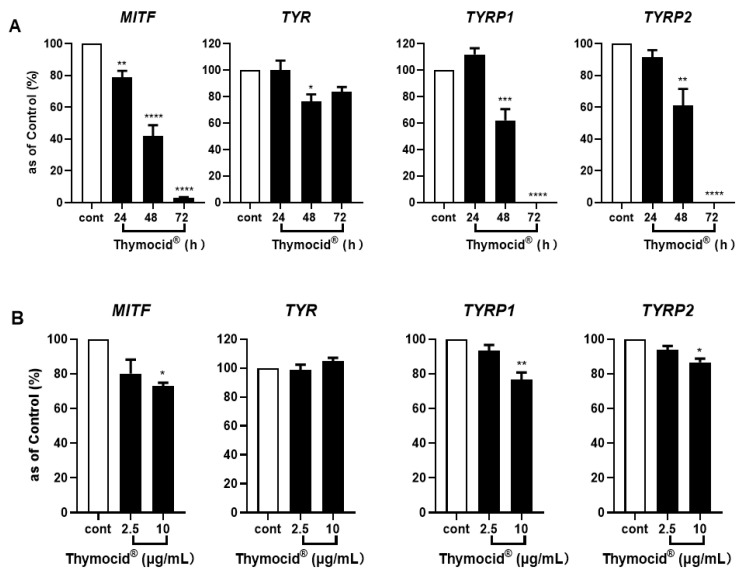
Effects of Thymocid^®^ on the expression of melanogenesis-related genes and proteins in melanoma B16F10 cells. Cells were treated with Thymocid^®^ (20 µg/mL) for 24, 48, and 72 h and the expression of genes including *MITF*, *TYR*, *TYRP1*, and *TYRP2* were determined by real-time qPCR assay (**A**). B16F10 cells were treated with Thymocid^®^ (2.5 and 10 µg/mL) for 72 h, and the expression of genes including *MITF*, *TYR*, *TYRP1*, and *TYRP2* were determined by real-time qPCR (**B**). *B2M* was used as an internal control in real-time qPCR assay. Significance was defined as * *p* < 0.05, ** *p* < 0.01, *** *p* < 0.005, **** *p* < 0.001 when compared to the control group. Values are presented as the means ± S.D. from three experimental replicates.

To further investigate the mechanism of Thymocid^®^’s suppression of melanogenesis (i.e., melanin production) in B16F10 cells, its effects on the expression of melanogenesis-related genes including *MITF*, *TYR*, *TYRP1*, and *TYRP2* were evaluated. As shown in Figure 5A, treatment of B16F10 cells with Thymocid^®^ (at 20 µg/mL) for 24, 48, or 72 h, suppressed the mRNA expression of *MITF*, *TYR*, *TYRP1*, and *TYRP2* in a time-dependent manner. At 48 and 72 h, treatment with Thymocid^®^ had a suppressive effect on the expression of *MITF*, *TYR*, *TYRP1*, and *TYRP2* as it reduced their mRNA expression to 42.0%, 76.5%, 62.2%, and 61.2%, and 3.3%, 83.6%, 0.3%, and 0.2%, respectively, whereas it only reduced expression of *MITF* to 78.9% within 24 h (Figure 5A). Moreover, we observed that treatment with Thymocid^®^, at 2.5 and 10 µg/mL, resulted in a concentration-dependent modulation of mRNA expression of *MITF*, *TYR, TYRP1*, and *TYRP2* to 80.1%, 98.6%, 93.4%, and 93.7%, and 73.0%, 104.9%, 76.6%, and 86.6%, respectively (Figure 5B).

**Figure 6:**
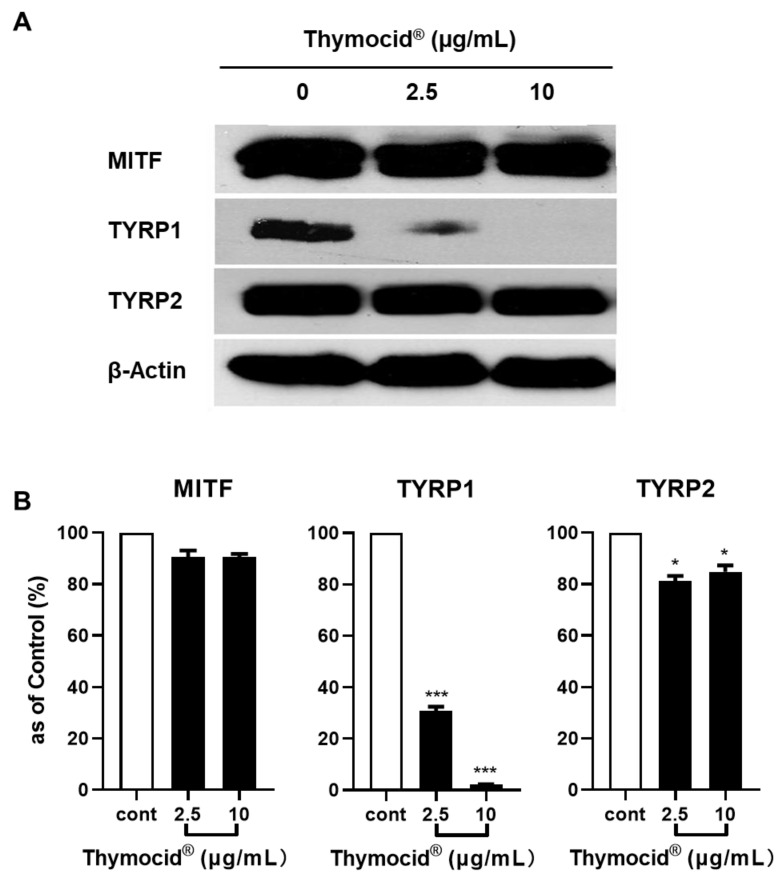
Effects of Thymocid^®^ on the expression of the melanogenesis-related proteins in B16F10 cells. Cells were treated with Thymocid^®^ (2.5 and 10 µg/mL) for 72 h and the expression of proteins including MITF, TYRP1, and TYRP2 were determined by Western blotting assay (densitometric data; **A**). Equal protein loading was confirmed by using protein β-actin as an internal household protein. The protein expression in B16F10 cells treated with Thymocid^®^ (2.5 and 10 µg/mL) for 72 h were compared to the control group (**B**). Values are presented as the means ± S.D. from three experimental replicates. Significance was defined as: * *p* < 0.05 and *** *p* < 0.001 when compared to the control group.

Next, we evaluated the effect of Thymocid^®^ on the expression levels of proteins related to melanogenesis in B16F10 cells by Western blotting assay. The densitometric data showed that treatment with Thymocid^®^ reduced the expression of TYRP1 and TYRP2 in B16F10 cells (Figure 6A). Quantitative analysis of data from Western blotting assay revealed that Thymocid^®^ (2.5 and 10 µg/mL) reduced suppressed the protein expression levels of TYRP1 (by 69.1–97.7%) and TYRP2 (by 9.4–9.3%), respectively (Figure 6B), whilst the expression of MITF was only slightly decreased by the treatment with Thymocid^®^ by 18.8–15.2% at 2.5 and 10 µg/mL, respectively) (Figure 6B).

## 4. Discussion

Thymoquinone (TQ), a major bioactive compound in BCSE [29], was used to standardize Thymocid^®^. TQ has been reported to show several skin beneficial effects such as chemoprevention of skin tumorigenesis [30] and anti-inflammatory effects against chemical-toxin-induced ear edema [30]. Although TQ may contribute to the overall skin beneficial effects of Thymocid^®^, other phytochemicals present in Thymocid^®^ may also exert biological effects in an additive, complementary, and/or synergistic manner. In fact, TQ showed effects similar to those of Thymocid^®^ in some bioassays, including anti-elastase and antityrosinase conducted in this study (Supplementary Materials Tables S1–S3); however, TQ at similar concentrations was less active than Thymocid^®^, suggesting that other phytochemicals present in Thymocid^®^ also contributed to its overall biological effects. In addition, although only one major peak appeared in the HPLC chromatogram of Thymocid^®^ monitored at the wavelength of 254 nm (retention time at 16.5 min; Figure 1), it is possible that other phytochemicals, including non-aromatic molecules, such as aliphatic compounds, may also be present in Thymocid^®^ without being detectable by HPLC analysis. Future studies to identify these aliphatic compounds using proper analytical tools (e.g., gas chromatography–mass spectrometry) and evaluate their biological activities are warranted. Moreover, the presence of other TQ derivatives including thymol, carvacrol, thymohydroquinone, and dithymoquinone, as well as alkaloids including nigellicine, nigellimine, nigellidine, and nigelanoid are also reported in published studies on the chemical composition of BCSEs [4,6]. Although compounds including carvacrol and thymol were not identified and quantified in Thymocid^®^ in the current study, it has been reported that carvacrol and thymol exhibited skin-protective effects including inhibitory effects on collagenase and elastase [31]. Therefore, it is possible that several phytochemicals including aliphatic compounds and alkaloids in Thymocid^®^ may also exert biological effects that contribute to the overall skin beneficial effects of BCSE. Therefore, a “whole-food” approach was used to evaluate the biological effects of Thymocid^®^, rather than testing its individual compounds. Thymocid^®^ showed anti-aging effects by maintaining protein structure against glycation (Figure 2). The inhibitory effect of Thymocid^®^ on the formation of AGEs in a BSA–fructose model (Figure 2A) was in agreement with a published study showing that TQ reduced the formation of AGEs in a model with human serum albumin and glucose [32]. In addition, a BCSE has been reported to show preventive effects against glycation-induced DNA damage [33]. Furthermore, Thymocid^®^ protected the structure of bovine type I collagen from glycation-induced cross-linking (Figure 2B). Given that the formation of AGEs is an oxidative process, it is possible that the antiglycation effect of Thymocid^®^ may attribute to its antioxidant effects [34]. The protective effects of Thymocid^®^ on the structure of skin’s connective proteins were supported by its anti-collagenase (Figure 3) and anti-elastase activities (Table 1). This is in agreement with a published study showing that a BCSE was able to inhibit the activity of human neutrophil elastase [35]. However, due to the structural and functional difference between porcine pancreatic elastase and human neutrophil elastase [36], further studies on Thymocid^®^’s effects on elastase in human skin fibroblast cells are warranted to support its antiwrinkle effects.

Melanin, the end-product of melanogenesis, is produced in melanocytes within melanosomes. It is a critical factor for the color of various organs and tissues including eyes, hair, and skin [37,38,39]. Melanin’s main role is related to skin protection against ultraviolet rays; however, excessive production and accumulation of melanin lead to cutaneous hyperpigmentation disorders, including freckles, skin discoloration, and pigmented age spots [38,40]. Over the years, this can lead to the enhancement of the degradation of cutaneous extracellular matrix proteins as observed in biological processes including skin aging, discoloration, and solar elastosis [41]. The process of melanogenesis is catalyzed by three major melanocyte-specific enzymes, namely, TYR, TYRP1, and TYRP2 (also known as dopachrome tautomerase) [40], with TYR being the rate-limiting enzyme for melanin biosynthesis [42,43,44]. These enzymes are appropriate targets for the improvement of skin conditions including skin tone, aging, whitening, or discoloration with plant-based nutrients [42,45]. Thymocid^®^ promoted the activity of tyrosinase, which corroborates a previously reported study showing that BCSE was able to increase melanin production in melanophores isolated from wall lizard (*Podarcis muralis*) [29]. However, further evaluations on the melanin content in murine melanoma B16F10 cells indicated that Thymocid^®^, at nontoxic concentrations, suppressed the production of melanin (Figure 4B,C) while it decreased cellular tyrosinase activity (Figure 4D). This is similar to a previously reported study, in which a BCSE was able to reduce melanin production in B16F10 cells stimulated with alpha-melanocyte-stimulating hormone [46]. The BCSE’s antimelanogenic effect (observed in this study and a study from another group) is contradictory with its melanogenic effect reported in a model of melanophores from wall lizard [30]. Several factors may account for this contradiction. First, BCSE’s effects on melanogenesis were assessed with different models (murine melanoma cells vs. wall lizard melanophores), which may have distinct response to the treatment with BCSE. Next, levels of TQ in BCSE used in the wall lizard melanophores study and in Thymocid^®^ are different (0.0356% vs. 5.12%). Therefore, further studies with more physiological relevant models including human melanocytes are warranted to confirm the modulatory effects of Thymocid^®^ on melanogenesis. Mechanistically, our studies showed that Thymocid^®^ inhibited cellular tyrosinase activity (Figure 4D) and suppressed the gene and protein expressions of melanogenesis-related markers in B16F10 cells (Figure 5 and Figure 6), which may collectively contribute to its overall antimelanogenic effects. We noted that Thymocid^®^ was able to downregulate both gene and protein expression of TYRP1 in B16F10 cells. TYRP1 is an enzyme catalyzing the oxidation of dihydroxyindole carboxylic acid during the process of melanin biosynthesis in murine melanoma cells [47]. In addition, TYRP1 is involved in maintaining the protein structure of tyrosinase and modulating its oxidative activity, which was in agreement with the observation of decreased cellular tyrosinase activity in B16F10 cells. Therefore, TYRP1 may be a valid molecular target for the antimelanogenic effects of Thymocid^®^. However, further studies with human melanocytes as well as in skin tissue architecture that simulate human skin are warranted to confirm this. In addition, further studies on Thymocid^®^’s cosmeceutical characterizations including skin permeability, bioavailability, and proper formulations are warranted.

## 5. Conclusions

In summary, Thymocid^®^, a chemically standardized and commercially available BCSE, protected the structure of BSA and type I collagen by inhibition of protein glycation and collagen cross-linking, respectively. Thymocid^®^ also inhibited the activity of skin-wrinkle-related enzymes including collagenase and elastase. The inhibitory effects of Thymocid^®^ on collagenase and elastase may contribute to its overall anti-aging effects. In addition, Thymocid^®^ showed inhibitory effects on the production of melanin content in B16F10 cells. This antimelanogenic effect was associated with its modulation of cellular tyrosinase activity and expressions of melanogenesis-related genes and proteins including MITF, TYR, TYRP1, and TYRP2. Our findings highlight the potential of Thymocid^®^ as a bioactive ingredient for cosmeceutical applications.

## Figures and Tables

**Table 1:** Inhibitory activity of Thymocid^®^ on elastase enzyme.

Sample	Concentration (µg/mL)	Inhibition Rate (%) ^a^	
		Type-I	Type-III
Thymocid^®^	1000	75.4 ± 4.5	91.1 ± 1.2
	500	69.2 ± 1.3	88.6 ± 2.3
	250	56.2 ± 3.2	78.4 ± 3.2
	125	35.4 ± 6.9	61.3 ± 2.1
	62.5	25.3 ± 5.9	36.0 ± 2.7
EGCG ^b^	92	73.0 ± 1.8	75.2 ± 4.0

^a^ Values are expressed as mean ± S.D. from three replicated experiments. ^b^ Epigallocatechin gallate; positive control.

**Table 2:** Modulatory effect of Thymocid^®^ on the activity of tyrosinase enzyme. Thymocid^®^ was evaluated with mushroom tyrosinase with two substrates including L-tyrosine and L-DOPA.

Sample	Concentration (µg/mL)	Enzyme Activity (%) ^a^	
		L-Tyrosine	L-DOPA
Thymocid^®^	1000	228.7 ± 9.6	146.3 ± 18.6
	500	192.0 ± 12.3	133.1 ± 6.1
	250	170.0 ± 6.8	133.9 ± 15.4
	125	162.5 ± 7.3	116.1 ± 6.7
	62.5	153.3 ± 6.6	113.7 ± 3.0
Kojic acid ^b^	10	49.5 ± 3.0	66.2 ± 13.2

^a^ Values are expressed as mean ± S.D. from three replicated experiments. ^b^ Positive control.

## Supplementary Materials

The following are available online at https://www.mdpi.com/2072-6643/12/7/2146/s1, Figure S1. HPLC chromatograms of Thymocid^®^, Figure S2. Standard curve for quantification of TQ in Thymocid^®^, Table S1. Inhibitory effect of TQ on collagenase activity, Table S2. Inhibitory effect of TQ on elastase activity, Table S3. Inhibitory effect of TQ on tyrosinase activity.

## Abbreviations

AG	aminoguanidine
AGEs	advanced glycation end-products
AAAPVN	N-succinyl-Ala-Ala-Ala-p-nitroanilide
BCSEs	black cumin seed extracts
BSA	bovine serum albumin
EGCG	epigallocatechin gallate
FALGPA	N-(3-[2-furyl]-acryloyl)-Leu-Gly-Pro-Ala
L-DOPA	L-3,4-dihydroxyphenylalanine
MGO	methylglyoxal
MITF	microphthalmia-associated transcription factor
phen	1,10-phenanthroine
TQ	thymoquinone
TYR	tyrosinase
TYRP1	tyrosinase-related protein-1
TYRP2	tyrosinase-related protein-2
