# Supplementary material for: Thymocid®, a Standardized Black Cumin (Nigella sativa) Seed Extract, Modulates Collagen Cross-Linking, Collagenase and Elastase Activities, and Melanogenesis in Murine B16F10 Melanoma Cells
Source: Nutrients. 2020 Jul 19;12(7):2146. doi: 10.3390/nu12072146 (PMC7400895; doi:10.3390/nu12072146)
Supplement: Supplementary file 1 [file nutrients-12-02146-s001.pdf]

Supplementary Materials

Thymocid®, A Standardized Black Cumin (*Nigella sativa*) Seeds Extract, Modulates Collagen Cross-Linking, Collagenase and Elastase Activities, and Melanogenesis in Murine B16F10 Melanoma Cells

Huifang Li [1,2], Nicholas A. DaSilva [2], Weixi Liu [3], Jialin Xu [2,4], George W. Dombi [3], Joel A. Dain [3], Dongli Li [1], Jean Christopher Chamcheu [5], Navindra P. Seeram [2], Hang Ma [1,2]*

[1] School of Biotechnology and Health Sciences, Wuyi University; International Healthcare Innovation Institute (Jiangmen), Jiangmen 529020, China; huifang_li@uri.edu (H.L.); wyuchemldl@126.com (D.L.)

[2] Bioactive Botanical Research Laboratory, Department of Biomedical and Pharmaceutical Sciences, College of Pharmacy, University of Rhode Island, Kingston, RI 02881, USA; NickDasilva91@gmail.com (N.A.D.); nseeram@uri.edu (N.P.S.); hang_ma@uri.edu (H.M.)

[3] Department of Chemistry, University of Rhode Island, Kingston, RI 02881, USA; weixi_liu@my.uri.edu (W.L.); gdombi@chm.uri.edu (G.W.D.); jdain@chm.uri.edu (J.A.D.)

[4] Institute of Biochemistry and Molecular Biology, College of Life and Health Sciences, Northeastern University, Shenyang 110819, China; jialin_xu@mail.neu.edu.cn (J.X.)

[5] School of Basic Pharmaceutical and Toxicological Sciences, College of Pharmacy, University of Louisiana at Monroe, Monroe, LA 71209, USA; chamcheu@ulm.edu (J.C.C.)

*Correspondence: hang_ma@uri.edu; Tel.: +1-401-874-7654

List of contents

Figure S1. HPLC chromatograms of Thymocid®……………………………………………………...…….3

Figure S2. Standard curve for quantification of TQ in Thymocid®…………………………………….……….4

Table S1. Inhibitory effect of TQ on collagenase activity………………………………………………….…....5

Table S2. Inhibitory effect of TQ on elastase activity…………………………….……….............……... 6

Table S3. Inhibitory effect of TQ on tyrosinase activity……………………………………..............……...7

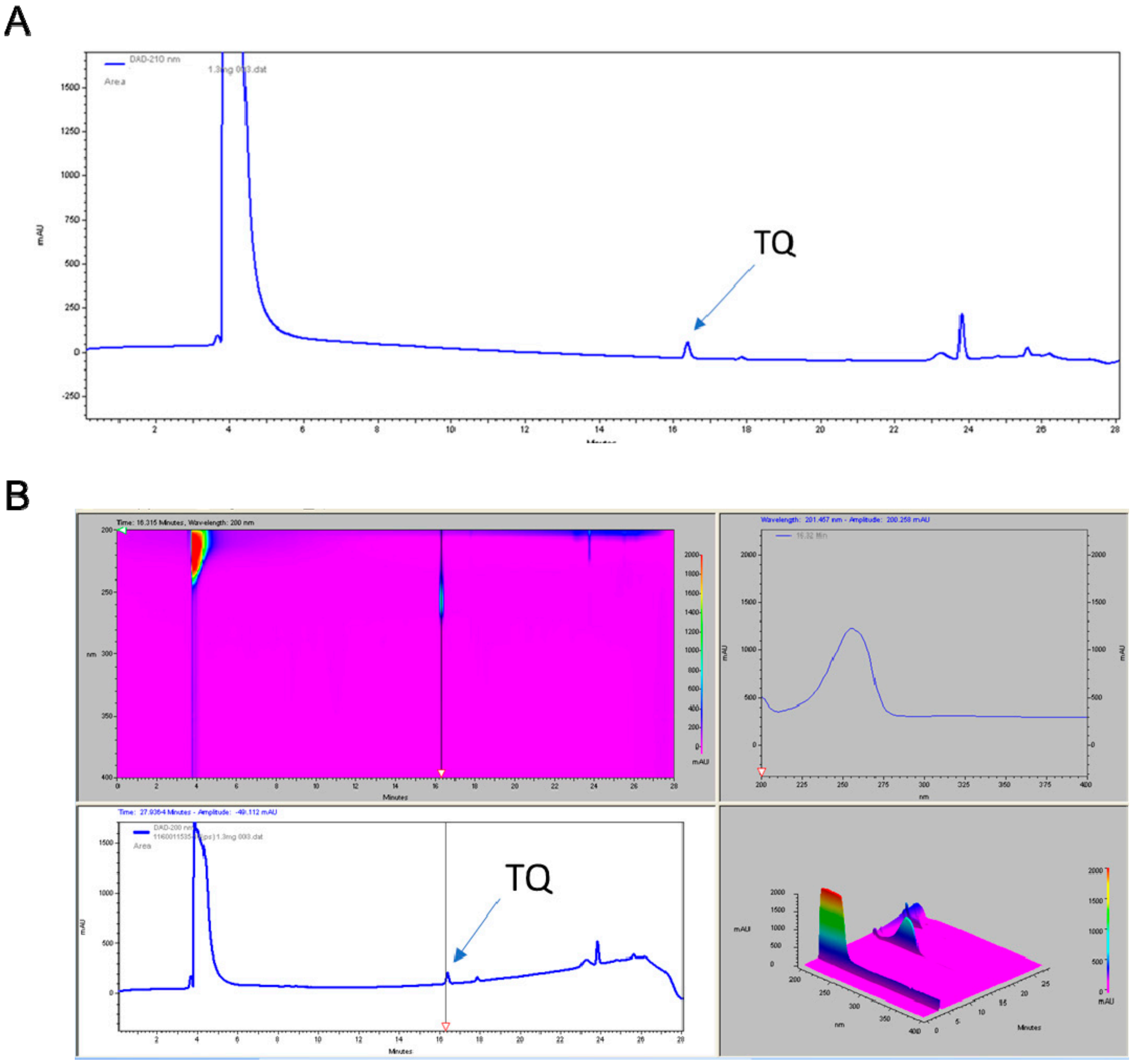

Figure S1. HPLC chromatograms of Thymocid® and TQ was monitored at the wavelength of 210 nm (A) and a mixed wavelength ranging from 200-400 nm (B)

Concentration (µg/mL)	AUC
1	126011
5	2106298
10	4472913
50	34805071
100	67556237

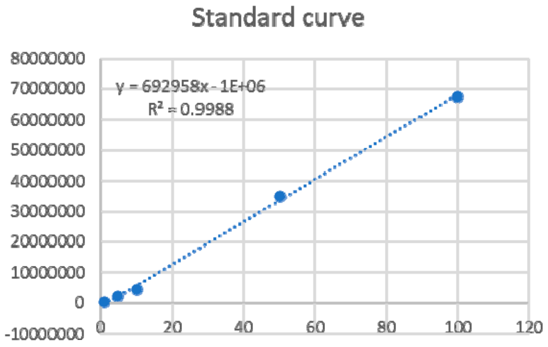

Figure S2. Standard curve used for the quantification of TQ in Thymocid®.

Table S1. Inhibitory activity of TQ on collagenase enzyme.

Sample	Concentration (µg /mL)	Enzyme activity (%)
TQ	1000	30.0 ± 7.6
	500	18.7 ± 5.0
	250	14.7 ± 7.1
	125	21.7 ± 11.1
	62.5	18.0 ± 5.7
phen[a]	10	98.4 ± 4.5

[a]Positive control.

Table S2. Inhibitory activity of TQ on elastase enzyme.

Sample	Concentration (μg /mL)	Inhibition rate (%)	
		Type-I	Type-III
TQ	1000	30.2 ± 0.1	45.3 ± 1.9
	500	27.1 ± 2.0	36.6 ± 5.3
	250	20.2 ± 2.8	26.9 ± 1.4
	125	18.1 ± 3.3	19.3 ± 5.3
	62.5	16.1 ± 1.0	12.9 ± 2.9
EGCG[a]	92	73.0 ± 1.8	75.2 ± 4.0

[a]Positive control.

Table S3. Inhibitory effect of TQ on tyrosinase enzyme.

Sample	Concentration (μg /mL)	Enzyme activity (%)	
		L-tyrosine	L-DOPA
TQ	1000	110.8 ± 1.7	117.7 ± 9.3
	500	113.3 ± 1.8	115.2 ± 6.3
	250	119.8 ± 9.7	123.8 ± 8.7
	125	124.5 ± 8.5	114.8 ± 16.5
	62.5	120.7 ± 8.6	100.8 ± 4.8
Kojic acid[a]	10	49.5 ± 3.0	66.2 ± 13.2

[a]Positive control.
